# What Is A “Difficult To Treat” Schizophrenia Patient

**DOI:** 10.1192/j.eurpsy.2023.952

**Published:** 2023-07-19

**Authors:** J. Petta, M. Nascimento, C. Oliveira, A. L. Falcão, G. Soares, A. Lourenço

**Affiliations:** Centro Hospitalar Psiquiátrico de Lisboa, Lisboa, Portugal

## Abstract

**Introduction:**

The Portuguese Plan for Mental Health envisaged the development of teams dedicated to the support of “difficult” patients. However, it was not clarified who these patients were, nor in which dimensions they could be supported. In this regard, there is a need for an objective and pragmatic definition to understand who these patients are.

**Objectives:**

To characterize the “difficult” patient with Schizophrenia.

**Methods:**

Through the hospital’s IT services, all acute inpatient episodes at Centro Hospitalar Psiquiátrico de Lisboa were collected since 2017, with the diagnosis of Schizophrenia (ICD10: F20 – n: 1448). Cluster analysis was performed, regarding number of previous admissions (PA) and days of admission. Descriptive analysis of these patients was made, regarding age, gender, destination at discharge, and to the “difficult to treat” patients, whether they attend a medical consultation prior to admission, if they were complying with the therapy and if they were using psychoactive substances.

**Results:**

Cluster analysis identified 3 clusters: (G1) a larger, uncharacteristic one; (G2) one of users with many PA; and one with a high number of days of admission (G3).

The average age is similar (46 years old), as well as gender (male). Regarding hospitalization days, G1 and G2 presented similar average values (16 days), higher for G3 (60 days). Comparing PA in G2, 47% of patients have between 6 and 10 PA and 25% have between 11 and 20 PA. For the same intervals, G3 has values of 10% and 2% respectively. About the destination after discharge, about 2/3 of both groups were referred for follow-up consultation; in G2, 5% were discharged by abandonment and in G3, 5% were referred to a Rehabilitation service and 6% integrated in Residential homes. Approximately 2/3 of the patients in G2 and G3 did not go to a medical consultation in the three months prior to their admission. Regarding the therapeutic plan, in G2 73% were not following it and in G3 this rate was 66%. Only 5% of G2 and 2% of G3 were in involuntary treatment. Injectable medication was used by 42% of patients in G2 and 23% in G3. Regarding substance use, alcohol was present in 9% of G2 and in 6% of G3; cannabinoids in 18% of G2 and in 11% of G3; and other psychoactive substances were present in 8% of G2 and in 4% of G3.

**Conclusions:**

The findings of this study allow us to outline two profiles of “difficult to treat” patients with Schizophrenia. On the one hand those with multiple relapses (G2), on the other those with prolonged hospitalizations (G3). Both have poor adherence to consultations and are erratic in therapeutic compliance. Injectable medication, although present in G2 and in a lower percentage in G3, and the infrequent involuntary treatment in both, may be considered as possible intervention points. An assertive multidisciplinary approach, focused on current treatment and relapse prevention (including social structures and rehabilitation centers), will be the key to their treatment.

**Disclosure of Interest:**

None Declared

